# Supplementary material for: Perception of rural adolescents and parents regarding child marriage: Findings of a community-based cross-sectional study in Bangladesh
Source: PLoS One. 2025 Aug 7;20(8):e0329648. doi: 10.1371/journal.pone.0329648 (PMC12331074; doi:10.1371/journal.pone.0329648)
Supplement: S1 Table — (DOCX) [file pone.0329648.s001.docx]

**Supplementary table 1: Correlation across statements (n=1030)**

| Attributes | Statistics | Social Phenomenon | Female Vulnerability | Interruption of education | Poor Social impact | Occurs due to parents’ decision | Reduced by adolescent involvement | Preventable by women empowerment | Permitted by Religion | Brings poor economic consequences | Imposing Poverty | Escaping Dowry | Economically beneficial | Perceives legal age | Burning Issue | Favors child marriage | Acceptable than late marriage | Helps getting better spouse | Poor Health consequences | Legal violation | Punishable Offence | Conjugal maladjustment | Domestic violence | Security in living environment | Prevents negative attitude of the society | Prevents premarital relations | Perception Score |
| --- | --- | --- | --- | --- | --- | --- | --- | --- | --- | --- | --- | --- | --- | --- | --- | --- | --- | --- | --- | --- | --- | --- | --- | --- | --- | --- | --- |
| Social Phenomenon | r | 1 | .039 | .014 | -.011 | .155** | .145** | .153** | -.037 | -.006 | .081** | .118** | .024 | .102** | .096** | -.006 | -.194** | -.147** | .069* | .033 | .019 | .044 | .041 | -.118** | -.088** | -.039 | .221** |
|  | Sig. |  | .212 | .656 | .719 | .000 | .000 | .000 | .233 | .859 | .010 | .000 | .437 | .001 | .002 | .840 | .000 | .000 | .027 | .287 | .535 | .157 | .186 | .000 | .005 | .214 | .000 |
| Female Vulnerability | r | .039 | 1 | .037 | .003 | -.024 | -.037 | -.025 | .033 | .019 | -.024 | .021 | .030 | .018 | .095** | .016 | -.060 | .026 | -.009 | -.008 | -.019 | -.028 | -.032 | .005 | -.030 | -.001 | .062* |
|  | Sig. | .212 |  | .232 | .917 | .443 | .233 | .415 | .295 | .546 | .451 | .499 | .340 | .571 | .002 | .611 | .054 | .404 | .770 | .791 | .533 | .373 | .305 | .872 | .344 | .967 | .047 |
| Interruption of education | r | .014 | .037 | 1 | .351** | .084** | .087** | .073* | .018 | .365** | .121** | .094** | .041 | .119** | .092** | .186** | .028 | -.001 | .524** | .207** | .205** | .378** | .373** | .068* | .038 | .007 | .415** |
|  | Sig. | .656 | .232 |  | .000 | .007 | .005 | .019 | .555 | .000 | .000 | .003 | .187 | .000 | .003 | .000 | .375 | .974 | .000 | .000 | .000 | .000 | .000 | .030 | .224 | .813 | .000 |
| Poor Social impact | r | -.011 | .003 | .351** | 1 | .017 | .184** | -.031 | .174** | .737** | .143** | .116** | .119** | .082** | .077* | .206** | .135** | .157** | .424** | .252** | .278** | .454** | .454** | .231** | .178** | .147** | .614** |
|  | Sig. | .719 | .917 | .000 |  | .575 | .000 | .315 | .000 | .000 | .000 | .000 | .000 | .009 | .014 | .000 | .000 | .000 | .000 | .000 | .000 | .000 | .000 | .000 | .000 | .000 | .000 |
| Occurs due to parents’ decision | r | .155** | -.024 | .084** | .017 | 1 | .206** | .073* | -.080* | .033 | .074* | .132** | -.009 | .047 | .021 | .002 | -.050 | -.029 | .121** | .040 | .065* | .070* | .056 | -.003 | -.005 | -.034 | .241** |
|  | Sig. | .000 | .443 | .007 | .575 |  | .000 | .020 | .010 | .292 | .017 | .000 | .784 | .132 | .502 | .949 | .111 | .354 | .000 | .196 | .036 | .024 | .074 | .932 | .878 | .277 | .000 |
| Reduced by adolescent involvement | r | .145** | -.037 | .087** | .184** | .206** | 1 | .051 | -.016 | .120** | .078* | .058 | .071* | .013 | .158** | .091** | -.029 | -.004 | .150** | .073* | .094** | .153** | .145** | -.034 | -.019 | -.095** | .309** |
|  | Sig. | .000 | .233 | .005 | .000 | .000 |  | .100 | .612 | .000 | .012 | .061 | .023 | .688 | .000 | .004 | .354 | .889 | .000 | .019 | .003 | .000 | .000 | .276 | .543 | .002 | .000 |
| Preventable by women empowerment | r | .153** | -.025 | .073* | -.031 | .073* | .051 | 1 | -.001 | -.001 | .040 | -.018 | -.005 | .037 | .089** | -.012 | -.077* | -.093** | .071* | .064* | .038 | .085** | .076* | -.048 | -.080* | -.093** | .184** |
|  | Sig. | .000 | .415 | .019 | .315 | .020 | .100 |  | .979 | .973 | .200 | .572 | .871 | .240 | .004 | .696 | .013 | .003 | .023 | .039 | .225 | .006 | .015 | .121 | .010 | .003 | .000 |
| Permitted by Religion | r | -.037 | .033 | .018 | .174** | -.080* | -.016 | -.001 | 1 | .144** | -.017 | -.065* | .113** | -.013 | .019 | .121** | .065* | .078* | .091** | .080* | .087** | .033 | .044 | .128** | .162** | .139** | .254** |
|  | Sig. | .233 | .295 | .555 | .000 | .010 | .612 | .979 |  | .000 | .596 | .036 | .000 | .668 | .545 | .000 | .036 | .012 | .004 | .010 | .005 | .295 | .156 | .000 | .000 | .000 | .000 |
| Brings poor economic consequences | r | -.006 | .019 | .365** | .737** | .033 | .120** | -.001 | .144** | 1 | .164** | .104** | .129** | .044 | .039 | .184** | .067* | .091** | .439** | .222** | .240** | .407** | .418** | .161** | .114** | .112** | .551** |
|  | Sig. | .859 | .546 | .000 | .000 | .292 | .000 | .973 | .000 |  | .000 | .001 | .000 | .162 | .205 | .000 | .032 | .004 | .000 | .000 | .000 | .000 | .000 | .000 | .000 | .000 | .000 |
| Imposing Poverty | r | .081** | -.024 | .121** | .143** | .074* | .078* | .040 | -.017 | .164** | 1 | .283** | -.101** | .049 | .044 | .067* | .039 | .008 | .148** | .101** | .098** | .137** | .118** | .021 | .009 | -.042 | .312** |
|  | Sig. | .010 | .451 | .000 | .000 | .017 | .012 | .200 | .596 | .000 |  | .000 | .001 | .119 | .162 | .033 | .217 | .802 | .000 | .001 | .002 | .000 | .000 | .502 | .781 | .181 | .000 |
| Escaping Dowry | r | .118** | .021 | .094** | .116** | .132** | .058 | -.018 | -.065* | .104** | .283** | 1 | -.016 | .095** | .007 | .026 | -.013 | -.015 | .063* | .047 | .068* | .134** | .092** | .060 | .003 | .004 | .323** |
|  | Sig. | .000 | .499 | .003 | .000 | .000 | .061 | .572 | .036 | .001 | .000 |  | .616 | .002 | .810 | .404 | .669 | .629 | .045 | .128 | .029 | .000 | .003 | .053 | .932 | .886 | .000 |
| Economically beneficial | r | .024 | .030 | .041 | .119** | -.009 | .071* | -.005 | .113** | .129** | -.101** | -.016 | 1 | .024 | .080* | .057 | -.014 | .109** | .039 | .024 | .058 | .064* | .095** | .080** | .128** | .067* | .232** |
|  | Sig. | .437 | .340 | .187 | .000 | .784 | .023 | .871 | .000 | .000 | .001 | .616 |  | .449 | .011 | .067 | .656 | .000 | .213 | .450 | .062 | .039 | .002 | .010 | .000 | .031 | .000 |
| Perceives legal age | r | .102** | .018 | .119** | .082** | .047 | .013 | .037 | -.013 | .044 | .049 | .095** | .024 | 1 | .087** | .040 | -.017 | .028 | .104** | .093** | .074* | .066* | .060 | .003 | -.035 | .002 | .237** |
|  | Sig. | .001 | .571 | .000 | .009 | .132 | .688 | .240 | .668 | .162 | .119 | .002 | .449 |  | .005 | .194 | .595 | .363 | .001 | .003 | .018 | .033 | .055 | .932 | .266 | .960 | .000 |
| Burning Issue | r | .096** | .095** | .092** | .077* | .021 | .158** | .089** | .019 | .039 | .044 | .007 | .080* | .087** | 1 | .065* | .039 | .022 | .130** | .088** | .078* | .171** | .131** | -.044 | .025 | .014 | .244** |
|  | Sig. | .002 | .002 | .003 | .014 | .502 | .000 | .004 | .545 | .205 | .162 | .810 | .011 | .005 |  | .037 | .208 | .478 | .000 | .005 | .012 | .000 | .000 | .160 | .422 | .645 | .000 |
| Favors child marriage | r | -.006 | .016 | .186** | .206** | .002 | .091** | -.012 | .121** | .184** | .067* | .026 | .057 | .040 | .065* | 1 | .187** | .094** | .323** | .210** | .170** | .224** | .213** | .118** | .136** | .065* | .325** |
|  | Sig. | .840 | .611 | .000 | .000 | .949 | .004 | .696 | .000 | .000 | .033 | .404 | .067 | .194 | .037 |  | .000 | .002 | .000 | .000 | .000 | .000 | .000 | .000 | .000 | .037 | .000 |
| Acceptable than late marriage | r | -.194** | -.060 | .028 | .135** | -.050 | -.029 | -.077* | .065* | .067* | .039 | -.013 | -.014 | -.017 | .039 | .187** | 1 | .414** | .094** | .044 | .016 | .113** | .121** | .327** | .302** | .228** | .298** |
|  | Sig. | .000 | .054 | .375 | .000 | .111 | .354 | .013 | .036 | .032 | .217 | .669 | .656 | .595 | .208 | .000 |  | .000 | .003 | .163 | .607 | .000 | .000 | .000 | .000 | .000 | .000 |
| Helps getting better spouse | r | -.147** | .026 | -.001 | .157** | -.029 | -.004 | -.093** | .078* | .091** | .008 | -.015 | .109** | .028 | .022 | .094** | .414** | 1 | .069* | .072* | .066* | .072* | .081** | .454** | .392** | .391** | .399** |
|  | Sig. | .000 | .404 | .974 | .000 | .354 | .889 | .003 | .012 | .004 | .802 | .629 | .000 | .363 | .478 | .002 | .000 |  | .027 | .021 | .035 | .021 | .009 | .000 | .000 | .000 | .000 |
| Poor Health consequences | r | .069* | -.009 | .524** | .424** | .121** | .150** | .071* | .091** | .439** | .148** | .063* | .039 | .104** | .130** | .323** | .094** | .069* | 1 | .312** | .299** | .421** | .416** | .084** | .063* | .025 | .509** |
|  | Sig. | .027 | .770 | .000 | .000 | .000 | .000 | .023 | .004 | .000 | .000 | .045 | .213 | .001 | .000 | .000 | .003 | .027 |  | .000 | .000 | .000 | .000 | .007 | .044 | .422 | .000 |
| Legal violation | r | .033 | -.008 | .207** | .252** | .040 | .073* | .064* | .080* | .222** | .101** | .047 | .024 | .093** | .088** | .210** | .044 | .072* | .312** | 1 | .794** | .240** | .243** | .065* | .055 | .029 | .386** |
|  | Sig. | .287 | .791 | .000 | .000 | .196 | .019 | .039 | .010 | .000 | .001 | .128 | .450 | .003 | .005 | .000 | .163 | .021 | .000 |  | .000 | .000 | .000 | .038 | .077 | .347 | .000 |
| Punishable Offence | r | .019 | -.019 | .205** | .278** | .065* | .094** | .038 | .087** | .240** | .098** | .068* | .058 | .074* | .078* | .170** | .016 | .066* | .299** | .794** | 1 | .256** | .226** | .113** | .066* | .056 | .403** |
|  | Sig. | .535 | .533 | .000 | .000 | .036 | .003 | .225 | .005 | .000 | .002 | .029 | .062 | .018 | .012 | .000 | .607 | .035 | .000 | .000 |  | .000 | .000 | .000 | .033 | .074 | .000 |
| Conjugal maladjustment | r | .044 | -.028 | .378** | .454** | .070* | .153** | .085** | .033 | .407** | .137** | .134** | .064* | .066* | .171** | .224** | .113** | .072* | .421** | .240** | .256** | 1 | .873** | .131** | .055 | .031 | .561** |
|  | Sig. | .157 | .373 | .000 | .000 | .024 | .000 | .006 | .295 | .000 | .000 | .000 | .039 | .033 | .000 | .000 | .000 | .021 | .000 | .000 | .000 |  | 0.000 | .000 | .076 | .323 | .000 |
| Domestic violence | r | .041 | -.032 | .373** | .454** | .056 | .145** | .076* | .044 | .418** | .118** | .092** | .095** | .060 | .131** | .213** | .121** | .081** | .416** | .243** | .226** | .873** | 1 | .137** | .071* | .040 | .552** |
|  | Sig. | .186 | .305 | .000 | .000 | .074 | .000 | .015 | .156 | .000 | .000 | .003 | .002 | .055 | .000 | .000 | .000 | .009 | .000 | .000 | .000 | 0.000 |  | .000 | .022 | .198 | .000 |
| Security in living environment | r | -.118** | .005 | .068* | .231** | -.003 | -.034 | -.048 | .128** | .161** | .021 | .060 | .080** | .003 | -.044 | .118** | .327** | .454** | .084** | .065* | .113** | .131** | .137** | 1 | .500** | .465** | .487** |
|  | Sig. | .000 | .872 | .030 | .000 | .932 | .276 | .121 | .000 | .000 | .502 | .053 | .010 | .932 | .160 | .000 | .000 | .000 | .007 | .038 | .000 | .000 | .000 |  | .000 | .000 | .000 |
| Prevents negative attitude of the society | r | -.088** | -.030 | .038 | .178** | -.005 | -.019 | -.080* | .162** | .114** | .009 | .003 | .128** | -.035 | .025 | .136** | .302** | .392** | .063* | .055 | .066* | .055 | .071* | .500** | 1 | .523** | .452** |
|  | Sig. | .005 | .344 | .224 | .000 | .878 | .543 | .010 | .000 | .000 | .781 | .932 | .000 | .266 | .422 | .000 | .000 | .000 | .044 | .077 | .033 | .076 | .022 | .000 |  | .000 | .000 |
| Prevents premarital relations | r | -.039 | -.001 | .007 | .147** | -.034 | -.095** | -.093** | .139** | .112** | -.042 | .004 | .067* | .002 | .014 | .065* | .228** | .391** | .025 | .029 | .056 | .031 | .040 | .465** | .523** | 1 | .408** |
|  | Sig. | .214 | .967 | .813 | .000 | .277 | .002 | .003 | .000 | .000 | .181 | .886 | .031 | .960 | .645 | .037 | .000 | .000 | .422 | .347 | .074 | .323 | .198 | .000 | .000 |  | .000 |
| Perception Score | r | .221** | .062* | .415** | .614** | .241** | .309** | .184** | .254** | .551** | .312** | .323** | .232** | .237** | .244** | .325** | .298** | .399** | .509** | .386** | .403** | .561** | .552** | .487** | .452** | .408** | 1 |
|  | Sig. | .000 | .047 | .000 | .000 | .000 | .000 | .000 | .000 | .000 | .000 | .000 | .000 | .000 | .000 | .000 | .000 | .000 | .000 | .000 | .000 | .000 | .000 | .000 | .000 | .000 |  |

r= Pearson’s correlation coefficient
