# Supplementary material for: Perception of rural adolescents and parents regarding child marriage: Findings of a community-based cross-sectional study in Bangladesh
Source: PLoS One. 2025 Aug 7;20(8):e0329648. doi: 10.1371/journal.pone.0329648 (PMC12331074; doi:10.1371/journal.pone.0329648)
Supplement: S2 Table — (DOCX) [file pone.0329648.s002.docx]

**Supplementary Table 2: Correlations across domains**

| **Scores** | **Statistics** | **Social domain score** | **Economic domain score** | **Psychological domain score** | **Environmental domain score** | **Total perception score** |
| --- | --- | --- | --- | --- | --- | --- |
| **Social domain score** | r | 1 | .309^**^ | .226^**^ | .162^**^ | .655^**^ |
|  | Sig. |  | .000 | .000 | .000 | .000 |
| **Economic domain score** | r | .309^**^ | 1 | .233^**^ | .235^**^ | .606^**^ |
|  | Sig. | .000 |  | .000 | .000 | .000 |
| **Psychological domain score** | r | .226^**^ | .233^**^ | 1 | .458^**^ | .693^**^ |
|  | Sig. | .000 | .000 |  | .000 | .000 |
| **Environmental domain score** | r | .162^**^ | .235^**^ | .458^**^ | 1 | .727^**^ |
|  | Sig. | .000 | .000 | .000 |  | .000 |
| **Total perception score** | r | .655^**^ | .606^**^ | .693^**^ | .727^**^ | 1 |
|  | Sig. | .000 | .000 | .000 | .000 |  |

r= Pearson Correlation coefficient
