# Supplementary material for: Perception of rural adolescents and parents regarding child marriage: Findings of a community-based cross-sectional study in Bangladesh
Source: PLoS One. 2025 Aug 7;20(8):e0329648. doi: 10.1371/journal.pone.0329648 (PMC12331074; doi:10.1371/journal.pone.0329648)
Supplement: Attachment — Submitted filename: Review.docx [file pone.0329648.s005.docx]

**Summary:**

In this study, the authors assess the perception of child marriage among adolescents and parents, focusing on differences in perceptions across the two groups. They rely on a primary survey of 1030 participants residing in four sub-district administrative units in rural Bangladesh. The cross-sectional survey involved face-to-face interviews using a semi-structured questionnaire that aimed to collect information on respondents’ demographic and socio-economic characteristics as well as their responses to 25 statements that measured their perception towards child marriage on four dimensions- social, economic, psychological and environmental. The authors conduct a multinomial logistic regression to determine the factors influencing perceptions towards child marriage. This was complemented by chi-square analysis to test if the differences in perceptions across each of the attributes considered are statistically significant. The authors present two key results. Firstly, they find that perception towards child marriage differs significantly across individuals with different level of education, current marital status and type of family (joint or nuclear) while socio-economic status, occupation, religion and gender do not explain differences in perception towards child marriage. Secondly, the authors find that adolescents and parent perceptions on social, economic and psychological attributes of child marriage do not differ significantly, whereas perceptions regarding environmental attributes of child marriage differ significantly across the two groups.

The study deals with a relevant social issue that persists in the context of the study- Bangladesh, as well as in other developing countries. It is important to understand how individuals perceive child marriage and to identify factors that drive support towards a practice that has been empirically established to have negative socio-economic outcomes for women, and the society at large. The authors adopt an appropriate method to this inquiry by using a semi-structured interview to gather data on this sensitive topic as it offers important insights into individual perceptions which are often difficult to interpret. Collecting sensitive data from a sample of 1030 individuals, along with useful demographic and socio-economic characteristics provides for a rich source of data. I also appreciate the safeguards the authors have adopted in carrying out this survey. By ensuring the surveys are conducted in anonymity and in the absence of other family members, they prevent some potential bias in responses.

However, the paper is not without its shortcomings. The analysis could be developed further to throw light onto the interactive effects of the various socio-economic characteristics and differences across adolescents and parental perceptions. The objective of the study and therefore, its implications, could be made more nuanced and clarified. The authors do not explain in enough detail how the attributes were chosen and classified into the four domains used in the analysis. Similarly, while there is some discussion on the factors that drive the observed perceptions, it is not clear where these conclusions are drawn from. I discuss these shortcomings and suggestions for improvement below. **Taking these into consideration, I recommend that the paper be re-assessed for publication after the authors have made major revisions to the current manuscript.**

**Major Revisions:**

1. Abstract/Introduction: The abstract describes the main objective of the study to be to “assess the perception of rural adolescents and parents regarding child marriage”. However, from the introduction and the discussion of the results, it is evident that the study focusses sufficiently on a comparison of differences in perceptions across adolescents and parents. The research question and objective need to be clarified to highlight a) that the study aims to compare differences in perceptions on child marriage across adolescents and parents; b) that the objectives include identifying factors/characteristics that influence perceptions on child marriage
2. Sample selection: I recommend adding a note on the representative of the unions of rural Bangladesh. There is some discussion towards the end (in the last session) but it would be appropriate to discuss the same earlier on.
3. The steps undertaken to survey the respondents has been discussed in great detail. However, certain methodological approaches need further explanation. Currently, there is no discussion on why the four domains- social, economic, psychological and environmental have been chosen. Moreover, the definition of each of these domains, especially with regards to child marriage needs to be included. More details on how the 25 questions/attributes were chosen and classified into the four domains would help the readers understand the concepts better. I am especially finding it difficult to distinguish between the attributes classified under social and environmental domains.
4. Page 10: Lines 235-244- I understand the responses to the statements were coded on a binary scale. However, attributing “correct answers” to 1 and “wrong answers” to 0 is not straightforward. Firstly, with the statements being subjective in nature, I do not think it is appropriate to classify them as correct and wrong answers. A more scientific approach would be to rank responses on a standardized scale where a higher value indicates support against child marriage. I would also recommend including the logistic regression specification in the manuscript.
5. Throughout the paper, the terms “good” and “bad” perceptions of child marriage have been used. It would be better to define these terms to ensure that readers understand that “good perception” as referred to in the paper implies support against child marriage.
6. In the discussion of the results, the authors refer to dowry, social norms etc. as reasons for the unanimous support in favor for child marriage prevalent in the sample. However, there is no explanation to how these conclusions were drawn. Are these conclusions drawn from evidence from open ended questions included in the survey? If so, please include details of the same. In case open-ended questions or any form of narrative analysis was undertaken, please include more details of how this was carried out, how surveyors recorded these responses and how the analysis was carried out.
7. I infer that the two main take-aways from this paper are that certain characteristics such as education, family structure and marital status can explain differences in perceptions on child marriage, and that perceptions on how child marriage affects environmental attributes differ across adolescents and parents. I would highlight these findings with further clarity in the conclusion. Moreover, I think it is possible to conduct further analyses to explore the intersection of these two aspects. For instance, it would be interesting to see if perceptions differ across adolescents and adults, given a level of education or gender.
8. It might be useful to include a discussion of potential confounders of perception of child marriage. For example, it is possible that surveyed parents who were themselves subjected to child marriage might harbor different perceptions than those who did not. Distinguishing/controlling for these factors would help identify factors that affect differential perceptions better. It might also be worth conducting further analyses after distinguishing between parents with sons and daughters. Would expect to see differences in perceptions between the two sets of parents but it would be interesting to see if the factors that drive perceptions differ across them.
9. The introduction and conclusion emphasize the importance of including the negative effects of child marriage in school curriculum to help change perceptions. Although this might have the desired effects, there is no evidence directly arising out of the analysis in this paper that suggests this. I recommend avoiding drawing such conclusions that are not directly linked to the evidence arising out of the study. Similarly, on page 17 (lines 337-340), the authors imply that social pressure is driving child marriages in Bangladesh. However, it is not clear if the questionnaire dealt with questions of social validation. I recommend the authors refer to the evidence supporting this statement or refrain from making them.

**Minor Revisions:**

1. More citations required:
   a. Page 3, Line 72
   b. Page 4, Line 94-95
   c. Page 5, line 114
   d. Page 6, Line 141
2. Page 4, line 98: Prevalence of child marriage has been adequately discussed. I would recommend balancing this discussion out with more evidence from the literature on the negative effects of child marriage on women’s outcomes and socio-economic outcomes, in general.
3. Page 5: The discussion of religion as a mediating factor for individual perceptions can be clarified further. The hypothesis seems to be that religion is an important mediating factor but the discussion needs to be refined to highlight how religion is expected to affect perceptions.
4. Page 6: Lines 133-143- can be moved to the introduction where the prevalence of child marriage in developing countries is discussed. Could also consider expanding on this discussion of the negative consequences of child marriage and highlight how this is motivates the current study.
5. Page 19: Line 369- “son-in-law” to be changed to “daughter-in-law”
