# Supplementary material for: Perception of rural adolescents and parents regarding child marriage: Findings of a community-based cross-sectional study in Bangladesh
Source: PLoS One. 2025 Aug 7;20(8):e0329648. doi: 10.1371/journal.pone.0329648 (PMC12331074; doi:10.1371/journal.pone.0329648)
Supplement: Attachment — Submitted filename: Response to Reviewers.docx [file pone.0329648.s007.docx]

**Perception of rural adolescents and parents regarding child marriage: Findings of a community-based cross-sectional study in Bangladesh**

**Journal Requirements:**

Please include the following items when submitting your revised manuscript:

- A rebuttal letter that responds to each point raised by the academic editor and reviewer(s). You should upload this letter as a separate file labeled 'Response to Reviewers'.
- A marked-up copy of your manuscript that highlights changes made to the original version. You should upload this as a separate file labeled 'Revised Manuscript with Track Changes'.
- An unmarked version of your revised paper without tracked changes. You should upload this as a separate file labeled 'Manuscript'.
- If you would like to make changes to your financial disclosure, please include your updated statement in your cover letter. Guidelines for resubmitting your figure files are available below the reviewer comments at the end of this letter.

**Responses:** Thanks for the valuable instructions. All the items are included with the submission

[1. Please ensure that your manuscript meets PLOS ONE's style requirements, including those for file naming.](1. Please ensure that your manuscript meets PLOS ONE's style requirements, including those for file naming. The PLOS ONE style templates can be found at https://journals.plos.org/plosone/s/file?id=wjVg/PLOSOne_formatting_sample_main_body.pdf and )

**Responses:** Thanks for the vital direction. The manuscript meets all PLOS ONE's style requirements

2. You indicated that you had ethical approval for your study. In your Methods section, please ensure you have also stated whether you obtained consent from parents or guardians of the minors included in the study or whether the research ethics committee or IRB specifically waived the need for their consent.

**Responses:** Thanks for the valuable guidelines. It is stated in the Methods section

3. We note that you have indicated that there are restrictions to data sharing for this study. PLOS only allows data to be available upon request if there are legal or ethical restrictions on sharing data publicly.

**Responses:** Thanks for the valuable instruction. It is stated that data will be available from the corresponding author upon reasonable request

**Responses to Comments of Reviewer # 1**

Comment 1. The authors investigate potential differences in attitudes towards child marriage between parents and adolescents who participated in the study. This is an interesting angle. However, the Background/Introduction section should provide a rationale for why examining these differences in perception and attitudes is important to explore.

Response: Thanks for the crucial comment. The rationale for examining the difference between adolescents and parents in perception regarding child marriage is provided at background section on pages 8-9. It is important to identify the differences to formulated adolescent and parent specific interventions through understanding their viewpoints developed on the basis of their knowledge and experiences.

Comment 2. A recent paper examines differences in attitudes towards child marriage in the context of an edutainment intervention. The authors may want to reference this article.

Islam, Muhammed Nazmul, Atonu Rabbani, Animesh Talukder, Rubaiya Riya Siddiqua, Sanjana Nujhat, Mushfiqur Rahman, Antara Roy, and Malabika Sarker. “Edutainment and the prevention of under-age marriages: The evaluation of a television series designed to promote positive role models in Bangladesh.” Journal of Development Effectiveness 16, no. 2 (2024): 159-186.

Response: Thanks for the vital comment. This article is referred in the discussion section at page 31 to exemplify possible interventions for improving the perception of parents and adolescents regarding child marriage.

Comment 3. The study uses a survey questionnaire to understand perceptions of child marriage. It would be helpful to explain the basis for these questions. Did the authors adopt any pre-existing tools? While all domains seem reasonable, what is the theoretical justification for them? I recommend linking the questions to relevant literature to provide justification and a foundation for the survey instrument.

Response: Thanks for the valuable comment. The data collection tool which included 25 statements to determine child marriage related perception was developed by the authors by extensive literature review and obtaining experts’ opinion. During data analysis phase, these statements were grouped in to 4 domains by sing thematic analysis [Data collection tool segment of Methods section, page-11]

Comment 4. Related to #3, the authors should elaborate on what the different domains mean and how questions across different domains relate to the scope of each specific domain.

Response: Thanks for the crucial comment. The meaning of each domain was operationalized by the authors and defined in Data collection tool segment of Methods section, page-11. the relation of each statement within each domain was analyzed by the authors with the help of experts.

Comment 5. Related to #4 (and #3), how did the researchers validate the questions?

Response: Thanks for the precious comment. Face validation of the tools was conducted by expert opinion. As we are not opting to build up a scale, this face validation could be enough as a validation process [Data collection tool segment of Methods section, pages 11-12].

Comment 6. In Table 3, what do "good," "average," and "poor" perception mean? Do they indicate how favorable respondents feel about child marriage outcomes?

Response: Thanks for the appreciated comment. Higher scores meaning good perception indication stronger support against child marriage, average perception means not so strong support against child marriage and poor perception means support for child marriage [Data processing and analysis section of methods, page 13].

Comment 7. P.11, lines 257-262 seem to repeat information from the Methods section. I recommend incorporating this into the Methods section without repetition.

Response: Thanks for the valuable comment. It is corrected accordingly.

Comment 8. The rows in Table 4 add up to 100% within each type of respondent. Isn’t reporting either "yes" or "no" sufficient?

Response: Thanks for the crucial comment. It could be done, the responses of ‘yes’ and ‘no’ are replaced by ‘agree’ and ‘disagree’ respectively. However, the same response like ‘Agree’ does not carry a specific value of 0 or 1 in all statements. To visualize the differences with more clarity, both agree and disagree frequency and percentage might be helpful.

Comment 9. The authors have not established expectations for the quantitative findings, making it difficult to interpret Table 4. In most cases, especially for the first three "domains," there doesn't seem to be much difference between the two types of respondents. Is this expected or not?

Response: Thanks for the vital comment. It is expected and can be explained by the same sources of knowledge regarding child marriage like schools and social media etc.

Comment 10. The only discernible differences appear in the "Environmental" domain. Why is this the case?

Response: Thanks for the valuable comment. This could be bi-chance or may be due to difference in live experience of family and society as parents have more experience than adolescents which is discussed in discussion section page-24.

Comment 11. Authors should report the correlation of answers across domains and questions. There should be tests for internal reliability of the survey tools.

Response: Thanks for the cherished comment. Correlation of answers across domains and questions were reported in supplementary tables 2 and 1 respectively showing significant correlation. Internal consistency was tested by Cronbach’s alpha test with obtained value of 0.68 [Data collection tool segment of Methods section, page-12].

Comment 12. The Discussion section primarily repeats the findings. This section should be used to suggest implications of the results and relate the findings to the broader literature. I request that the authors revise it accordingly.

Response: Thanks for the esteemed comment. The discussion section was revised accordingly.

**Responses to Comments of Reviewer # 2**

**Comments**: In this study, the authors assess the perception of child marriage among adolescents and parents, focusing on differences in perceptions across the two groups. They rely on a primary survey of 1030 participants residing in four sub-district administrative units in rural Bangladesh. The cross-sectional survey involved face-to-face interviews using a semi-structured questionnaire that aimed to collect information on respondents’ demographic and socio-economic characteristics as well as their responses to 25 statements that measured their perception towards child marriage on four dimensions- social, economic, psychological and environmental. The authors conduct a multinomial logistic regression to determine the factors influencing perceptions towards child marriage. This was complemented by chi-square analysis to test if the differences in perceptions across each of the attributes considered are statistically significant. The authors present two key results. Firstly, they find that perception towards child marriage differs significantly across individuals with different level of education, current marital status and type of family (joint or nuclear) while socio-economic status, occupation, religion and gender do not explain differences in perception towards child marriage. Secondly, the authors find that adolescents and parent perceptions on social, economic and psychological attributes of child marriage do not differ significantly, whereas perceptions regarding environmental attributes of child marriage differ significantly across the two groups.

The study deals with a relevant social issue that persists in the context of the study- Bangladesh, as well as in other developing countries. It is important to understand how individuals perceive child marriage and to identify factors that drive support towards a practice that has been empirically established to have negative socio-economic outcomes for women, and the society at large. The authors adopt an appropriate method to this inquiry by using a semi-structured interview to gather data on this sensitive topic as it offers important insights into individual perceptions which are often difficult to interpret. Collecting sensitive data from a sample of 1030 individuals, along with useful demographic and socio-economic characteristics provides for a rich source of data. I also appreciate the safeguards the authors have adopted in carrying out this survey. By ensuring the surveys are conducted in anonymity and in the absence of other family members, they prevent some potential bias in responses.

However, the paper is not without its shortcomings. The analysis could be developed further to throw light onto the interactive effects of the various socio-economic characteristics and differences across adolescents and parental perceptions. The objective of the study and therefore, its implications, could be made more nuanced and clarified. The authors do not explain in enough detail how the attributes were chosen and classified into the four domains used in the analysis. Similarly, while there is some discussion on the factors that drive the observed perceptions, it is not clear where these conclusions are drawn from. I discuss these shortcomings and suggestions for improvement below. Taking these into consideration, I recommend that the paper be re-assessed for publication after the authors have made major revisions to the current manuscript.

**Major Revisions:**

Comment 1. Abstract/Introduction: The abstract describes the main objective of the study to be to “assess the perception of rural adolescents and parents regarding child marriage”. However, from the introduction and the discussion of the results, it is evident that the study focusses sufficiently on a comparison of differences in perceptions across adolescents and parents. The research question and objective need to be clarified to highlight a) that the study aims to compare differences in perceptions on child marriage across adolescents and parents; b) that the objectives include identifying factors/characteristics that influence perceptions on child marriage.

Response: Thanks for the appreciated comment. Comparison in difference of perception between adolescents and parents and factors influencing perception regarding child marriage are added as objectives in both abstract [page-2] and in background [page 8&9].

Comment 2. Sample selection: I recommend adding a note on the representative of the unions of rural Bangladesh. There is some discussion towards the end (in the last session) but it would be appropriate to discuss the same earlier on.

Response: Thanks for the vital observation. A note on the representative of the unions of rural Bangladesh is added in sample size and sampling part of the methods on page 10.

Comment 3. The steps undertaken to survey the respondents has been discussed in great detail. However, certain methodological approaches need further explanation. Currently, there is no discussion on why the four domains- social, economic, psychological and environmental have been chosen. Moreover, the definition of each of these domains, especially with regards to child marriage needs to be included. More details on how the 25 questions/attributes were chosen and classified into the four domains would help the readers understand the concepts better. I am especially finding it difficult to distinguish between the attributes classified under social and environmental domains.

Response: Thanks for the valuable comment. Twenty-five statements were chosen by expert opinion and extensive literature review. The during data analysis, these statements were grouped in to four themes by thematic analysis by the researchers. The domains were defined and mentioned at ‘data collection section’ page-11.

Comment 4. Page 10: Lines 235-244- I understand the responses to the statements were coded on a binary scale. However, attributing “correct answers” to 1 and “wrong answers” to 0 is not straightforward. Firstly, with the statements being subjective in nature, I do not think it is appropriate to classify them as correct and wrong answers. A more scientific approach would be to rank responses on a standardized scale where a higher value indicates support against child marriage. I would also recommend including the logistic regression specification in the manuscript.

Response: Thanks for the valuable observation. Considering the subjectivity of answers, ‘yes’ and ‘no’ responses were replaced by ‘agree’ and ‘disagree’. Favourable response indicating support against child marriage was given 1 and unfavourable response was given 0 value. Higher value indicated strong support against child marriage [Data processing and statistical analysis section page, 13]. Logistic regression specification was added in page 13.

Comment 5. Throughout the paper, the terms “good” and “bad” perceptions of child marriage have been used. It would be better to define these terms to ensure that readers understand that “good perception” as referred to in the paper implies support against child marriage.

Response: Thanks for the vital observation. Corrected according to that [Data processing and statistical analysis section page- 13]

Comment 6. In the discussion of the results, the authors refer to dowry, social norms etc. as reasons for the unanimous support in favor for child marriage prevalent in the sample. However, there is no explanation to how these conclusions were drawn. Are these conclusions drawn from evidence from open ended questions included in the survey? If so, please include details of the same. In case open-ended questions or any form of narrative analysis was undertaken, please include more details of how this was carried out, how surveyors recorded these responses and how the analysis was carried out.

Response: Thanks for the vital comment. The difference of perception attributed by education, family type and marital status was mentioned in conclusion section. The difference of perception variance was more prominent in environmental domain also added in conclusion and the difference was likely due to difference of live experience between both groups [Page-32].

Comment 7. I infer that the two main take-away from this paper are that certain characteristics such as education, family structure and marital status can explain differences in perceptions on child marriage, and that perceptions on how child marriage affects environmental attributes differ across adolescents and parents. I would highlight these findings with further clarity in the conclusion. Moreover, I think it is possible to conduct further analyses to explore the intersection of these two aspects. For instance, it would be interesting to see if perceptions differ across adolescents and adults, given a level of education or gender.

Response: Thanks for the crucial observation. The difference of perception between adolescents and parents across different sociodemographic characteristics like gender, education, family and marital status are shown in Table -4, page 18. It showed significant difference between both groups with primary level education and married participants.

Comment 8. It might be useful to include a discussion of potential confounders of perception of child marriage. For example, it is possible that surveyed parents who were themselves subjected to child marriage might harbor different perceptions than those who did not. Distinguishing/controlling for these factors would help identify factors that affect differential perceptions better. It might also be worth conducting further analyses after distinguishing between parents with sons and daughters. Would expect to see differences in perceptions between the two sets of parents but it would be interesting to see if the factors that drive perceptions differ across them.

Response: Thanks for the vital observation. The marital age of the parents was not included in this study hence the perception difference between parents subjected to child marriage and adult marriage could not be compared. This limitation was included in discussion section page [Page -32]

Comment 9. The introduction and conclusion emphasize the importance of including the negative effects of child marriage in school curriculum to help change perceptions. Although this might have the desired effects, there is no evidence directly arising out of the analysis in this paper that suggests this. I recommend avoiding drawing such conclusions that are not directly linked to the evidence arising out of the study. Similarly, on page 17 (lines 337-340), the authors imply that social pressure is driving child marriages in Bangladesh. However, it is not clear if the questionnaire dealt with questions of social validation. I recommend the authors refer to the evidence supporting this statement or refrain from making them.

Response: Thanks for the vital observation. The recommendations regarding inclusion of negative effects of child marriage in school curriculum was excluded from the manuscript. Regarding social pressure driving child marriage, references were mentioned in introduction [pages 6-7] and conclusion [pages 25-26].

**Minor Revisions:**

Comment 1. More citations required:

a. Page 3, Line 72- page 4, line [82-83]

b. Page 4, Line 94-95 - page 5 line [122-123]

c. Page 5, line 114 - page 6 line [144-146]

d. Page 6, Line 141- page 7 line [165-166]

Response: More citations added in above mentioned pages

Comment 2. Page 4, line 98: Prevalence of child marriage has been adequately discussed. I would recommend balancing this discussion out with more evidence from the literature on the negative effects of child marriage on women’s outcomes and socio-economic outcomes, in general.

Response: Discussion regarding negative effects of child marriage was added after the prevalence of child marriage mentioned in introduction section at page 5-6.

Comment 3. Page 5: The discussion of religion as a mediating factor for individual perceptions can be clarified further. The hypothesis seems to be that religion is an important mediating factor but the discussion needs to be refined to highlight how religion is expected to affect perceptions.

Response: The role of religion to affect social norms and human perception is mentioned in manuscript. However, the role of single particular religion is not a established as followers of different religion in various countries were found to be more inclined to child marriage- found by systematic review in South-east Asia [Page 6-7].

Comment 4. Page 6: Lines 133-143- can be moved to the introduction where the prevalence of child marriage in developing countries is discussed. Could also consider expanding on this discussion of the negative consequences of child marriage and highlight how this is motivates the current study.

Response: These lines are moved after the prevalence of child marriage in developing countries were discussed [page-5].

Comment 5. Page 19: Line 369- “son-in-law” to be changed to “daughter-in-law”

Response: Son in law is replaced by grandchildren at page 27 in economic dimension of discussion section.
