# Supplementary material for: Perception of rural adolescents and parents regarding child marriage: Findings of a community-based cross-sectional study in Bangladesh
Source: PLoS One. 2025 Aug 7;20(8):e0329648. doi: 10.1371/journal.pone.0329648 (PMC12331074; doi:10.1371/journal.pone.0329648)
Supplement: Attachment — Submitted filename: Child Marriage_Responses to Review Comments_R.2.docx [file pone.0329648.s008.docx]

**Perception of rural adolescents and parents regarding child marriage: Findings of a community-based cross-sectional study in Bangladesh**

**Responses to Review Comments**

**Key points requiring revision**

1. **Terminology Clarity**: The use of terms such as “poor” or “good” perceptions remain ambiguous. The reviewer strongly recommends replacing these with clearer terms such as “support” or “do not support” child marriage, to avoid misinterpretation.

**Response:** Thank a lot for the valued comment. Three categories of perception level; ‘good, ‘average’ and ‘poor’ have been replaced by two categories as “support” and “do not support” following your suggestion.

2. **Conceptual Framing of Statements**: The classification of survey items into domains needs further clarification. It is important to distinguish between statements about causes, consequences, and value judgments. Some statements currently included may reflect observations rather than normative views.

**Response:** Thanks for the pertinent comment. The concept behind four domains is described in details at Page-12, lines: 279-292. Some survey statements are rephrased to distinguish them clearly as cause, consequence or value judgement as applicable in Table-3 (pages-17-18) and Table-5 (pages-19-20).

At present, it is not possible to make all statements as normative views. Through extensive literature review, we found that most of the statements taken in this regards were observations and opinions of the participants rather normative views. Some relevant references of literature are given below-

Wibowo HR, Ratnaningsih M, Goodwin NJ, Ulum DF, Minnick E. One household, two worlds: Differences of perception towards child marriage among adolescent children and adults in Indonesia. The Lancet Regional Health–Western Pacific. 2021 Mar 1;8.

Naghizadeh S, Mirghafourvand M, Mohammadi A, Azizi M, Taghizadeh-Milani S, Ganbari H. Knowledge and viewpoint of adolescent girls regarding child marriage, its causes and consequences. BMC women's health. 2021 Dec;21:1-0.

3. **Sampling and Context**: Greater detail is needed on how the four unions were selected. Additionally, while we understand the need to protect participant identity, the manuscript would benefit from indicating the broad geographic location (e.g., district or upazila) if permissible under ethical guidelines.

**Response:** Thanks for the crucial comment. Four unions were selected as study places using multistage systematic random sampling technique, which is described in page 9 and in the page 10 under Figure -1.

4. **Wealth Index Categorization**: The authors should clarify how wealth indices were constructed. If quintiles were used, it would be clearer to refer to them as such instead of using less precise terms like “middle class.”

**Response:** Thanks a lot for the vital comment. The construction process is described with greater details in the page no 13. Wealth quintiles are used to categorize the socioeconomic class and mentioned as such according to your recommendation.

5. **Statistical Presentation**: Table 4 should be revised to present, for each characteristic, the percentage of parents and adolescents who support child marriage, along with corresponding p-values. This would improve the interpretability and relevance of the findings.

**Response:** Thanks for the valued comment. Table 4 (Pages, 18-19), and now Table 7 (Pages, 22-23), has been presented as per your recommendation.

6. **Discussion Section**: The discussion currently includes several statistical reiterations that belong in the results section. The authors should streamline this section and focus on interpretation and implications. The reviewer also encourages more detailed consideration of intervention strategies tailored to parents and adolescents, as well as reflection on why adolescents may exhibit more favorable attitudes toward child marriage than parents.

**Response:** Thanks a lot for the most crucial comment. In this current revised version, discussion section contains specific interpretation of the results with greater and detailed focus on implementation and intervention strategies for parents and adolescents

7. **Additional Points**: Minor issues such as missing publication years in references should also be corrected.

**Response:** Publication year is being corrected in reference list.

**Responses to Comments of the Reviewer #1:** 

The authors have provided itemized responses to the comments received in the first round. I have several more suggestions that I believe will help the authors to improve the manuscript.

1. I apologize but I still find the terms “poor” perception towards child marriage ambiguous. Why not use the terms such as “Least Favorable” views towards child marriage which is much easy interpret?

**Response:** Thank a lot for the valued comment. Three categories of perception level; ‘good, ‘average’ and ‘poor’ have been replaced by two categories as “support” and “do not support” following reviewer’s suggestion.

2. This is related to a comment I had earlier about the justifying the relevance of the perceptions towards early child marriage. Aren’t there prior works that show how perceptions towards a social phenomenon such as early marriage determine actual outcomes?

**Response:** Thanks a lot for your valued comment. In page 35 (line-725-733) we have described this issue and referred the findings of a community-level clustered randomized controlled trial in Bangladesh reporting the effect of addressing social norms favoring child marriage through tripping point intervention. The study found reduction in child marriage occurrence among the participants with higher attendance to the intervention sessions.

3. Page 10, lines 240-1: How did the researchers choose four unions to carry out the study? Is the present research based on another existing or ongoing research?

Thanks for the crucial comment. Four unions were selected as study places using multistage systematic random sampling technique, which is described in page 9 and in the page 10 under Figure -1.

4. Authors of the study also did not reveal the location of the study. Was it restricted by the IRB? Obviously, we don’t want the identities of the individual participants. But sharing the broad locations (district or upazila) can help the readers to contextualize the findings. Authors should consider this.

Thanks for your valuable suggestion. The study was conducted at Dhamrai upazila under the jurisdiction of Dhaka district.

5. Page 14, lines 340-3: How were the wealth indexes categorized? Were the divided into quintiles? If so, the authors should just use quintiles instead of using less well-defined terms such as “middle class.”

Thanks a lot for the vital comment. The construction process is described with greater details in the page no 13. Wealth quintiles are used to categorize the socioeconomic class and mentioned as such according to your recommendation.

6. Do the perceptions of the parents towards early child marriage vary by the sex of their children?

Thanks for your precious comment. The issue is addressed in table 5 page-19 and 20 revealing no significant difference in perception regarding child marriage among parents based on the sex of their children.

7. The authors repeat and mention lots of statistics in the discussion section. Many of them are redundant. They should be in the Findings section and authors should am to interpret the results more succinctly in the Discussion section. This will also help shortening the Discussion section.

Thanks a lot for the most crucial comment. In this current revised version, discussion section contains specific interpretation of the results with greater and detailed focus on implementation and intervention strategies for parents and adolescents

8. [Minor comment] Page 7, lines 171-8: The authors referenced to a paper without the publication year. Authors may want to revise the sentence.

**Response:** Publication year is being corrected in reference list.

**Responses to Comments of the Reviewer #2:** 

1. The statement selection into the domains is unconvincing. Although there is more clarity on what the different domains are in the revised version, I think it is important to distinguish between statements that refer to causes and consequences of child marriage. Moreover, some statements such as "child marriage is a burning issue", "occurs due to parents" etc. simply indicate what respondents think is happening and agreeing to these statements may not necessarily mean they do not support child marriage.

**Response:** Thanks for the pertinent comment. The concept behind four domains is described in details at Page-12, lines: 279-292. Some survey statements are rephrased to distinguish them clearly as cause, consequence or value judgement as applicable in Table-3 (pages-17-18) and Table-5 (pages-19-20).

At present, it is not possible to make all statements as normative views. Through extensive literature review, we found that most of the statements taken in this regards were observations and opinions of the participants rather normative views. Some relevant references of literature are given below-

Wibowo HR, Ratnaningsih M, Goodwin NJ, Ulum DF, Minnick E. One household, two worlds: Differences of perception towards child marriage among adolescent children and adults in Indonesia. The Lancet Regional Health–Western Pacific. 2021 Mar 1;8.

Naghizadeh S, Mirghafourvand M, Mohammadi A, Azizi M, Taghizadeh-Milani S, Ganbari H. Knowledge and viewpoint of adolescent girls regarding child marriage, its causes and consequences. BMC women's health. 2021 Dec;21:1-0.

2. Although you mention what "good" and "bad" perceptions are as used in the manuscript, I strongly advise you to change the terminology throughout the manuscript to "not support" and "support" child marriage. A good perception regarding child marriage could be mistaken to mean they perceive child marriage to be good and this is confusing for the reader.

**Response:** Thank a lot for the valued comment. Three categories of perception level; ‘good, ‘average’ and ‘poor’ have been replaced by two categories as “support” and “do not support” following your suggestion.

3. Thank you for adding Table 4 in response to the previous comments on intersectionality. In its current form, Table 4 is telling us what percent of those who support (or not) child marriage are adults and adolescents for each characteristic. Why aren’t the p-values from the chi-square test not reported for each group then? It might be more insightful if you can rework this table to show for each given characteristic, what percent of parents and adolescents supported child marriage and if this difference is significant. For instance, among those with secondary education, how many adolescents and parents perceived child marriage an issue (rather than looking at what percent of those with secondary education and reported "good perception" were adolescents and parents).

**Response:** Thanks for the valued comment. Table 4 (Pages, 18-19), and now Table 7 (Pages, 22-23), has been presented as per your recommendation.


4. In the discussion section, authors could include more implications from the study. Since a large part of the paper looks into differences in perceptions among 2 groups, who should the proposed awareness interventions target? Are there specific topics that need to be dealt with differently for either groups? How do the authors explain why the adolescents harbor more favorable stance in support of child marriage than parents?

**Response:** Thanks a lot for your valuable comment. In line with your suggestion, we have added more implication based on previous study findings. We also mentioned about group specific interventions which can be helpful for improving perception towards child marriage in this version. We found that adolescents and parents did not differ significantly in terms of overall and domain specific perception regarding child marriage. However, they differ significantly in some individual statements. In some statements, parents and in others adolescents showed favorable stance for child marriage.
