# Supplementary material for: Perception of rural adolescents and parents regarding child marriage: Findings of a community-based cross-sectional study in Bangladesh
Source: PLoS One. 2025 Aug 7;20(8):e0329648. doi: 10.1371/journal.pone.0329648 (PMC12331074; doi:10.1371/journal.pone.0329648)
Supplement: Attachment — Submitted filename: Child Marriage_Responses_Review Comments.docx [file pone.0329648.s009.docx]

**Journal Requirements:**

Please review your reference list to ensure that it is complete and correct. If you have cited papers that have been retracted, please include the rationale for doing so in the manuscript text, or remove these references and replace them with relevant current references. Any changes to the reference list should be mentioned in the rebuttal letter that accompanies your revised manuscript. If you need to cite a retracted article, indicate the article’s retracted status in the References list and also include a citation and full reference for the retraction notice.

Reference no 43 has been moved to 14

**Responses to Comments of Reviewer #1**

**Reviewer #1:** Comments on “Perception of rural adolescents and parents regarding child marriage: Findings of a community-based cross-sectional study in Bangladesh”

**Comment 1.** In Table 3, the agreement and disagreement always add to 100 percent in each row (as they should). Do we need both columns then? How about only agreement and showing the differences between the two columns? This suggestion applies to Table 5 as well.

**Response:** Thanks a lot for your valuable comment. Table 3 and 5 have been modified according to your recommendation.

**Comment 2**. In Table 4, the columns for parents and adolescents identical. Is there a mistake?

**Response:** Thanks a lot for your crucial query. The domain specific median (IQR) score for parents and adolescents were the same.

**Comment 3.** Figure 3 does not bode well aesthetically. Why use 3D bars? Please consider making it simpler, preferably making it in greyscale to make it printer friendly.

**Response:** Thanks a lot for your precise comment. Figure 2 has been constructed in greyscale as 2D bars

**Comment 4.** The authors should check the grammar and spelling of the manuscript more carefully. Is “Tripping Point Intervention” correct? I believe it is “Tipping”, not “Tripping.”

**Response:** Thanks you for in-depth observation. The spelling mistake is corrected in line 731, page-35

**Comment 5.** The authors should add an AI statement to the manuscript.

**Response:** Thanks a lot for your time-bound observation**.** As per your learned recommendation, AI statement has been added.

**Responses to Comments of Reviewer #2**

**Comment 1.** Stick to the same terminology throughout the paper: change “positive perception” usages in the sampling and sample size section (line 250-251, for example).

**Response:** Thanks for your learned opinion. The term “positive perception” has been changed to “supportive perception” in the specific areas.

**Comment 2.** P1 and P2 seem to be calculated ex-post and therefore cannot be used as sample size calculation parameters. If supported by other evidences, please discuss the same. If not, the sample size calculation can be moved to the appendix and presented as ex-post power calculation rather than ex-ante.

**Response:** Thank you for your relevant comment. The reference of p_1_ p_2_ values is provided correctly [25] at page no 10 line 251.

**Comment 3.** Remove unsubstantiated claims in the discussion section: For example, lines 476-478 are not supported with adequate citations.

**Response:** Lines 476-478 have been removed from the manuscript.

**Comment 4.** Highlight the differences in perception among adolescents and parents in the environment domain. If youngsters support for CM cannot be fully explained, discuss the puzzle and propose what policy can do to help in this case.

**Response:** Thank you for your precise comment. In page 32, line 641-643, it is mentioned that unlike other domains, both participant groups showed significant difference perception in all items of environmental domains. It is notable that, adolescents did not show significantly higher support for CM in all items of environmental domain rather in some items, parents showed significant higher support. For instance, significantly higher proportion of parents supported CM marriage in items like social insecurity, negative attitude of the society, premarital relationship etc. Adolescents sowed higher support in items like legal and familial issues associated with CM and possible explanation could be lack of practical life experience of such consequences. Appropriate interventions to inform these harmful consequences of CM using academic, and mass medial platforms are recommended [Page: 32, line :655-660]

While revising your submission, please upload your figure files to the Preflight Analysis and Conversion Engine (PACE) digital diagnostic tool, [https://pacev2.apexcovantage.com/](https://track.editorialmanager.com/CL0/https:%2F%2Fpacev2.apexcovantage.com%2F/1/010f0197ab41c1a9-e920c344-20c5-4c2e-8da4-ca45af5f3f07-000000/Yg91ebdC8FcI7uszqD4iJ21aUn-Md31QztjG2YB1VTE=217). PACE helps ensure that figures meet PLOS requirements. To use PACE, you must first register as a user. Registration is free. Then, login and navigate to the UPLOAD tab, where you will find detailed instructions on how to use the tool. If you encounter any issues or have any questions when using PACE, please email PLOS at [figures@plos.org](mailto:figures@plos.org). Please note that Supporting Information files do not need this step.

**Response:** Thanks a lot for the valuable suggestion. Figure files are uploaded to the Preflight Analysis and Conversion Engine (PACE) digital diagnostic.
